# Methods of Micropatterning and Manipulation of Cells for Biomedical Applications

**DOI:** 10.3390/mi8120347

**Published:** 2017-11-29

**Authors:** Adrian Martinez-Rivas, Génesis K. González-Quijano, Sergio Proa-Coronado, Childérick Séverac, Etienne Dague

**Affiliations:** 1 CIC, Instituto Politécnico Nacional (IPN), Av. Juan de Dios Bátiz S/N, Nueva Industrial Vallejo, 07738 Mexico City, Mexico; 2 CONACYT-CNMN, Instituto Politécnico Nacional (IPN), Av. Luis Enrique Erro s/n, Nueva Industrial Vallejo, 07738 Mexico City, Mexico; gkgonzalez@conacyt.mx; 3 ENCB, Instituto Politécnico Nacional (IPN), Av. Wilfrido Massieu, Unidad Adolfo López Mateos, 07738 Mexico City, Mexico; sergio.prc81@gmail.com; 4 ITAV-CNRS, Université de Toulouse, CNRS, Toulouse, France; childerick.severac@itav.fr; 5 LAAS-CNRS, Université de Toulouse, CNRS, Toulouse, France

**Keywords:** cell patterning and manipulation, mammalian and bacterial cells, micro-nanofabrication, microfluidics, organs-on-chips (OOC), biomedical microelectromechanical systems (bioMEMS), point-of-care (POC), soft lithography

## Abstract

Micropatterning and manipulation of mammalian and bacterial cells are important in biomedical studies to perform in vitro assays and to evaluate biochemical processes accurately, establishing the basis for implementing biomedical microelectromechanical systems (bioMEMS), point-of-care (POC) devices, or organs-on-chips (OOC), which impact on neurological, oncological, dermatologic, or tissue engineering issues as part of personalized medicine. Cell patterning represents a crucial step in fundamental and applied biological studies in vitro, hence today there are a myriad of materials and techniques that allow one to immobilize and manipulate cells, imitating the 3D in vivo milieu. This review focuses on current physical cell patterning, plus chemical and a combination of them both that utilizes different materials and cutting-edge micro-nanofabrication methodologies.

## 1. Introduction

The objective of micropatterning and manipulating mammalian and bacterial cells is to have better controls, a deeper understanding, and to apply these in practical biomedical microelectromechanical systems (bioMEMS), point-of-care (POC) devices, and organs-on-chips (OOC) [1]. In this regard, (nano)biotechnologists have developed and implemented novel methodologies to fix cells on substrates, in a controlled manner, so-called micropatterning. It is a challenging task, however, new micro and nanofabrication methodologies have contributed to the achievement of satisfactory outcomes. Cell micropatterning and cell manipulation currently represent the basic steps to perform drug testing experiments [2,3], to understand biochemical processes [4,5], to design microfluidic devices for medical applications, and to conduct fundamental studies in biological areas [6,7]. In this context, in vitro assays have increased their efficiency because of the simplicity of cell micropatterning and manipulation, which permit the carrying out of 3D human cells assays, replacing animal in vivo models [8]. Additionally, because of the versatility of these cell micropatternings, they can be applied to biomolecules [9], bacteria [10], yeasts [11], and other bioparticles involved in therapies [12], diagnosis [13], or interaction with numerous biochemical processes [14].

Single-cell manipulating models allow more in depth studies of membrane functionalities, cell interaction with particles, as well as drugs and external stimulus that a few years ago would have been difficult to analyze, including the advantage of performing high throughput measurements [15]. On the other hand, parallel-cell manipulation enables cell-arrays to mimic in vivo conditions, representing enormous progress in biomedical areas due to the fact that the conventional 2D culture is being replaced by 3D approaches which are more accurate and nearer to humans, both physiologically and metabolically [16]. 

It is straightforward to consider characteristics of the substrate where cells are patterned, such as conductivity, hydrophobicity, hydrophilicity, thermal, and environmental factors together with cost, and accessibility [17]. In this context, predominant substrates or platforms are composed of polymers such as polydimethylsiloxane (PDMS), polymethylmethacrylate (PMMA), cyclic olefin copolymer (COC), and polyimide (PI), while other biomaterials are gaining popularity such as alginate, chitosan, or functionalized surfaces with the use of low cost materials like graphene [18]. 

In this review, separated or combined physical and chemical techniques for micropatterning and manipulating mammalian and bacterial cells are described, focusing on microfabricated biomedical devices and surveying significant reported articles as well as the contributions of the present authors, in this area.

## 2. Techniques and Methods

### 2.1. Physical Cell Patterning

#### 2.1.1. Inkjet Cell Printing

Inkjet bioprinting uses an ink solution to generate droplets containing cells. There are three types of inkjet printing methodologies known as: continuous inkjet (CIJ) printing, drop-on demand (DOD) printing, and electrohydrodynamic jet printing. As there is high controllability and less contamination; the DOD inkjet printing method has been largely used to fabricate 3D structures for biomedical applications. Hence, Yusof et al. [19] reported a non-contact approach to pattern single cells by using an Inkjet printing technique that consisted of a dispenser chip to deposit droplets, a sensor to detect the cells, and an automation tool to print on specific substrates such as microscope slides and microtiter plates. They put emphasis on diagnostic and therapeutic applications by patterning a cervical cancer line (HeLa), obtaining a printing efficiency of 87% and a cell viability rate of 75%. This technique has also been used to implement 3D micro-tissue arrays [20], and then 440 micro-arrays or 3D liver tissue chips with different layer numbers and hepatocytes and endothelial cells were elaborated, as part of organ-on-chips developments. 

The DOD technique to elaborate live-cell-based biosensors has also been explored [21]. In this work the concentration of reactive oxygen species (ROS) was studied, as this is thought to be related to the change of hydrogen peroxide (H_2_O_2_) and then implicated in some human health conditions, including aging. A Surface Patterning Tool (SPT) substrate made of SU-8 resist was elaborated. They placed mammalian cells and modified a commercial Bioforce Nano eNablerTM (Bioforce Nanosciences, Inc., Ames, IA, USA), to print onto a hydrogel-based anchoring matrix. Inkjet printing has a moderate cost and good controllability; however, some parameters related to droplet formation had to be considered. Then in a recent paper [22] a ligament flow of a droplet formation process was obtained, when patterning cells and their effect on the viability and distribution were studied.

In another paper, an electrohydrodynamic jet printing (e-jet) approach was employed [23] to print bioinks such as fibronectin (FN), extracellular matrix (ECM) glycoprotein, and 3-aminopropyltriethoxysilane (APTES) to subsequently pattern mouse embryonic fibroblast cells (NIH-3T3). This methodology uses a rapid nozzle-free jet process called pyroelectrohydrodynamic jet (p-jet), because it uses a pyroelectric effect that modifies the bioink fluid, modulating the dot sizes from 200 μm down to 0.5 μm. 

**Figure 1:**
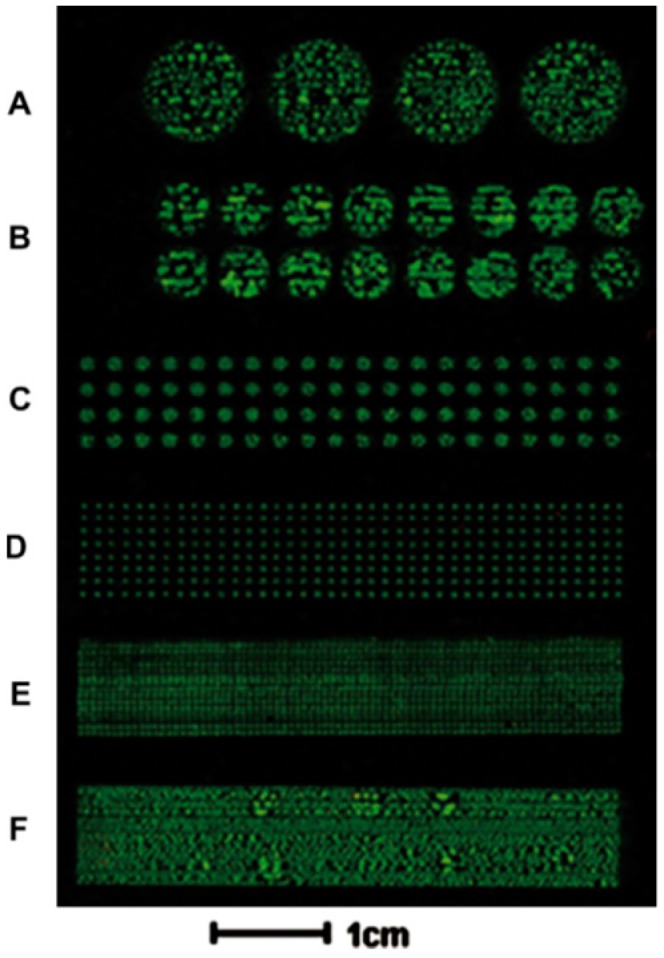
Microscope glass slide where a bacterial array was printed, showing different dot sizes in the letters **A** to **F**. Reproduced with permission from [24].

Relating to pattering of bacteria, Zheng et al. [24], modified a commercial thermal inkjet printer to pattern *Escherichia coli* on agar-coated substrates, making different bacterial colonies which enabled the evaluation of the antimicrobial activity of antibiotics. To perform the printing of bacteria onto microscope glass slides and microtiter plates, a commercial four-color thermal inkjet printer (Canon PIXMA ip1880) was employed (Figure 1). Srimongkon et al. [25] elaborated a prototype of a bacterial culture system by combining commercial inkjet printers and paper substrates to pattern cells in a culture media based on hydrogels such as poly(vinyl alcohol) and standard calcium alginate, as an alternative to the commonly used agarose.

#### 2.1.2. Optical and Optoelectronic Tweezers

This technology uses optical forces to move cells, some optical tweezers use radiation pressure emitted by a laser beam and others use infrared lasers. Cell arrays using optical methods allow remote manipulation and monitoring due to the intrinsic charge and dielectric properties of cells.

Ozkan et al. [26] fabricated an electro-optical system which employed both an electrophoretic array and remote optical manipulation by vertical-cavity surface. They were able to monitor the expression of a fluorescent protein in aseptic conditions. 

Optical tweezers provide high precision of positioning for small arrays and small dielectric objects. However, they have a limited manipulation area which means that at large-scale and heterogeneous patterns, the resolution is reduced [26,27]. To reduce optical radiation forces, optoelectronic methodologies can be applied to trap cells. Optoelectronic tweezers (OET) can reduce energy 100,000 times compared with optical tweezers as mentioned by Chiou et al. [28] when used with a halogen lamp and a digital micromirror for parallel manipulation of cells that were trapped on a 1.3 × 1.0 mm^2^ area with direct optical imaging control. They placed cells between an upper indium tin oxide-coated glass (ITO-coated glass), and lower multiple layers of photosensitive surfaces. This technique utilizes high-resolution virtual electrodes for single-cell manipulation and direct imaging to control live human B-cells and differentiates between dead cells, according to the image obtained and their dielectric properties. In addition, this technique permits high-resolution patterning using electric fields with less optical intensity than optical tweezers, therefore the differences in permeability, capacitance, conductivity, internal conductivity, and size allow one to discriminate between live cells and dead cells. 

Furthermore, levels of radiation can reach ~10^7^ W/cm^2^ which could cause photodamage to cells (opticution) [29]. There are other variants such as plasmonic tweezers, and photonic crystal waveguides, however they are limited by heat generation and light intensity and could cause cell damage [30].

Non-contact optoelectronic manipulation can be applied for some bacteria that have high movability. Mishra et al. [29] used an electrokinetic technique to manipulate *Enterobacter aerogenes* that in suspension reach >20 µm/s. They proved the optical radiation effect, laser-induced heating, and the electric field on bacteria viability. The system consisted of parallel-plate ITO-coated transparent electrodes separated by a 100 μm spacer to form a microchannel, a 1064 nm laser projecting into the microchannel through a 40X lens, and dark field imaging of bacteria cells. They used 10% BSA to avoid unspecific adherence to the electrodes and an AC electric field. Their experiments demonstrated that optical radiation and laser-induced heating have negligible effect on cell membranes. However, high electric field strength ≥200 KV_pp_ (peak to peak voltage), the combination of laser-induced temperature, and electrothermal flow can accelerate the poration of cells after ~5 min. 

It is possible, by the use of OET, to reach large-scale parallel manipulation and low-intensity optical trapping. Jing et al. [30] proposed modulated light fields to trap mammalian, yeast, and *Escherichia coli* cells, on the surface of a two-dimensional photonic crystal. They fabricated a silicon photocrystal coated with parylene-C to planarize the surface and provide an adequate refractive index. Circular patterns were obtained by photolithography as parallel holes of 500 nm in depth. By using this methodology, they trapped different single cells at the pattern’s surface without compromising their viability. They also proved that the aperture number of the lens did not affect the effectiveness of cell trapping and their methodology could be applied to miniaturize devices used for several types of cells.

Optoelectronic manipulation of cells is a feasible option for cell trapping and elaboration of microfluidic devices, due to remote and large-scale manipulation. Currently microfabrication techniques are enlarging their applications. Nonetheless, thermal effects and photodamage of cells must be critical factors in designing experimental systems with this methodology. 

#### 2.1.3. Laser-Based Cell Patterning

Laser-based direct writing technique to pattern cells, uses a laser to transfer or propel cells from one source film (donor, ribbon or target) to a receiving or acceptor substrate. This technique could be divided as follows [31]: Laser-induced forward transfer (LIFT), absorbing film-assisted laser-induced forward transfer (AFA-LIFT), biological laser processing (BioLP), matrix-assisted pulsed laser evaporation direct writing (MAPLE DW), and laser-guided direct writing (LGDW). LIFT, AFA-LIFT, BioLP, and MAPLE DW have a similar configuration and nowadays these techniques are referred to as laser direct-write (LDW). Hence, LDW combined with rat mesentery culture tissue have been employed to reproducibly print breast cancer cells (MDA-MB-231 and MCF-7) and fibroblasts on this ex vivo tissue [32]. In this article, it was recently demonstrated that by using this bioprinting technique, it was possible to locally pattern breast cancer cell groups to characterize cell movements during the angiogenesis. 

The last laser-based cell pattering called laser-guided direct writing (LGDW), is a variation technique of the commonly used optical trap (laser tweezers, optical tweezers), capable of depositing cells on different matrices such as collagen or Matrigel, but is limited by the size of the specific cell [33]. LGDW is a technique that consists of guiding and propelling a stream of cells onto a target surface by using optical forces of a laser (700–1000 nm which is above the wavelength absorption of most proteins). It has been used to propel embryonic chick spinal cord cells of a distance of around 300 µm, through their culture medium, and deposited in an untreated glass coverslip (as target surface) [31]. A total of 76 cells were guided with an average deposition rate of 2.5 cells/min. To increase the distance of the cell guidance to the maximum of 7 mm, light was coupled into hollow optical fibers, verifying the cell viability. It was finally claimed that this technique, in comparison to laser tweezers, has the advantage of presenting a continuous stream of cells for deposition and a position precision of 1 µm, being adaptable to microfabrication methodologies.

#### 2.1.4. Acoustic Force Patterning

Acoustic methodologies use surface acoustic waves (SAWs) for microscale manipulation with less energy than optical and optoelectronic approaches. SAWs, made of electrodes, are excited at different frequencies and deposited onto piezoelectric substrates. Common frequencies to generate SAW wavelengths from 1 to 300 nm are around 10 to 1000 MHz [34]. In acoustic manipulation systems, the displacement resolution depends on the formed nodes and frequency because of the applied energy [35]. Most of the works have been focused on the reduction of time and energy required to pattern cells, conserving their functionality and viability as mentioned by Ding et al. [35]. Their system consisted of a lithium niobate (LiNbO_3_) piezoelectric substrate collocated asymmetrically between two orthogonal pairs of interdigitated transducers (IDTs), with an independent radiofrequency signal. The orthogonal array formed four nodes around a polydimethylsiloxane (PDMS)-based microchannel that allowed total control in the displacement area. They found that the power density required to manipulate 10 μm polystyrene beads was ~0.5 nW/μm^2^ for a particle, reaching velocities of ~30 μm/s at 18.5 MHz to 37 MHz. They patterned (letters) with bovine red blood cells and polystyrene beads, furthermore under the same conditions they immobilized a multicellular microorganism *Caenorhabditis elegans*, not finding significant generated heat. The viability of these cells did not deteriorate. 

Acoustic methods can be applied for 3D microsystems as well. Recently Nasser et al. [36] used self-assembled monolayers (SAMs) to align cardiomyocytes mixed with cardiac fibroblasts in an extracellular matrix-based gelatin methacryloyl (GelMA). The piezoelectric substrate was lithium niobate (LiNbO_3_) and slanted-finger interdigital transducers (SFITs) were fabricated. The alignment was obtained in less than 10 s. The cells conserved their functionality after 5–7 days, this indicated that this methodology is suitable to create 3D biomimetic structures for rapidly encapsulating cells.

The migration of cells, subjected to acoustic waves is called acoustophoresis, and is dependent on the physical properties of cells such as size, compressibility, and density but also on the viscosity and fluidic properties of the medium. Most of the cells have a positive acoustic contrast factor that implies an attraction to nodes [37]. The principle of acoustophoretic microdevices is the same, that is, a piezoelectric platform and IDTs are needed to produce SAWs to generate cell movements in a continuous flow due to the acoustic force. The design created by Ai et al. [37] to separate *Escherichia coli* (*E. coli*) from human peripheral blood mononuclear cells (PBMCs) in a silicon-based microchannel demonstrated that the pressure nodes created on the sidewalls of the microchannel were perpendicular to the piezoelectric base, and the biggest cells (PBMCs) were attracted to nodes which were separated in different outlets, with an efficacy of 95.65% in continuous flow.

An acoustic method does not compromise cell viability, it is a chemical free technique to manipulate cells with less energy compared with an optical method and it is a rapid contactless technique, nonetheless a previous simulation process with the corresponding mathematical models could predict the behavior of cells, improving the results as in other physical methods.

#### 2.1.5. Electrokinetic Forces (Dielectrophoresis)

Dielectrophoresis (DEP) is considered an active method of cell manipulation because it requires energy to move cells. This technique combines electrokinetic forces with hydrodynamic effects to achieve cell trapping or lead cells to specific areas without damaging them. A cell has polarization in the surrounding media caused by an electric field. A dipole moment is induced by the electric field thereby moving cells, and depending on their permittivity and the polarizability of the surrounding media, the cells can be attracted to the electric field in the direction of the gradient (positive) or repelled, opposite to the gradient (negative) [38,39]. These considerations are important because cells can be separated from a mixture, as a positive and negative charge at first approach and then selecting the appropriate frequency, cells can be separated into groups usually at frequencies between 10 kHz to 100 MHz [13]. Cells can also be separated using combined methods such as flow separation, field-flow fractionation (FFF) (by sedimentation, temperature or viscosity), and travelling-wave mechanisms [38]. However, the displacement of cells becomes slow when the dielectric force decreases due to the separation distance of electrodes along the test area. To solve this problem, modern micro-nanofabrication techniques permit the elaboration of different geometries of nanometer-scale electrodes that can improve the area and the distribution of the electric field, using different arrays. These arrays have been widely used for medical microdevices. For example, Gascoyne et al. [13], detected malaria-infected cells from human blood by dielectrophoretic manipulation, using two types of microelectrode arrays. An interdigitated electrode array, operated at 5 V_pp_ and 200 kHz, was used to separate the parasitized erythrocytes by negative electrophoresis, and then a spiral electrode array operated with four-phase excitation at 3 V_pp_ and 2 MHz to concentrate parasitized erythrocytes at the center of the spiral was presented. In this particular case, parasitized erythrocytes, having pores due to the infection, they exhibited a loss of ions, changing their permittivity and membrane properties which facilitated their dielectric differentiation.

Actually, not only nanometer electrode arrays, and dielectrophoresis traps have improved cell manipulation, but control mechanisms have been integrated for this purpose. Recently, Sadeghian et al. [39] elaborated a microfluidic actuator with gold interdigitated electrode patterns to separate white blood cells (k562-cells) from polystyrene particles. They performed an optimization by finite element simulation in COMSOL Multiphysics 5, and according to their results, geometric parameters such as pitch, width to pitch ratio, and channel height are important because the gradient of the generated electric field depends on these factors. Efficiency of recovery was 93% with 100% of purity at 7.5 V_pp_ and 800 MHz. It was concluded that in their interdigitated electrode array the electrodes-pitch should be as close as possible. The channel must have a minimum height, and the voltage should be as high as possible but avoiding cell damage to achieve cell separation [39]. 

Furthermore, dielectrophoretic manipulation of cells has been applied to tissue engineering to align different types of cells in complex tissues as demonstrated by Ho et al. [40]; through positive dielectrophoresis (DEP), biomimetic alignment of lobular liver tissue was achieved, employing a concentric-stellate-tip electrode array that generates radial-pattern electric fields to guide individual cells. This cell-patterning microfluidic chip was fabricated on glass and PDMS, and planar electrodes were placed in a concentric ring array which provided the formation of pearl-chain like patterns. These patterns were stabilized because of the stellate-tip designed in the electrodes, which enhanced the distribution of the electric field with local maximum gradients inside the concentric-ring array, tangentially between the adjacent stellate-tip electrode rings.

**Figure 2:**
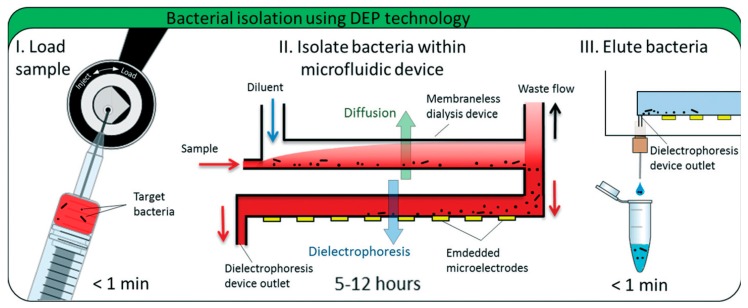
A microfluidic device to detect and separate pathogen bacteria from human blood. (**I**) Blood sample mixed with permeabilizing agent is loaded and injected, (**II**) The sample is pumped to the microfluidic device, (**III**) Target bacteria are eluted for further analysis. Reproduced with permission from [41].

Dielectric differentiation and manipulation could be applied not only to stem cells, cancer cells, and other biomolecules and particles associated with many pathologies, but also to microorganisms which may cause diseases in humans. D’Amico et al. [41] employed a co-planar quadrupole microelectrode geometry to detect low-levels of *Escherichia coli* and *Staphylococcus aureus* from human blood using a combined membraneless microdialysis and dielectrophoresis system. They isolated 79% of *E. coli* and 78% of *S. aureus* from minimal blood sample volumes. To reach bacteria separation, they used monensin to permeabilize blood cells and alter their cytoelectric properties to separate cells in their microfluidic system (Figure 2). This label-free methodology can be applied to detect other pathogens directly from biological samples reducing costs, time, instrumentation, cross contamination, and sample amounts through the microfabrication techniques for miniaturization procedures.

#### 2.1.6. Magnetic Cell Manipulation

**Figure 3:**
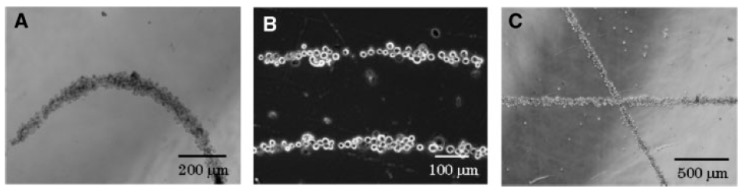
Phase-microscope images of cell patterns created by magnetic forces. (**A**) Fibroblasts (FB) curve patterns; (**B**) FB parallel patterns; (**C**) FB crossing patterns. Reproduced with permission from [42].

Magnetic force and magnetic biomaterials can guide cells for tissue engineering applications which require complex and functional tissue organization. Some have used magnetic manipulation to form patterns with complex architectures, Ino et al. [42] used magnetite cationic liposomes (MCLs) to label mouse NIH/3T3 fibroblasts (FB) and human umbilical vein endothelial cells (HUVECs) to form different patterns by using steel plates and a magnet (Figure 3). They proved variants such as cell patterning by laser-cut devices, and cell patterning of HUVECs onto Matrigel to create complex capillaries. Their results show non-toxic effects on cells, and very good formation of capillaries and branches by sequenced patterning.

Another novel system using magnetic force, was a 3D magnetic bioprinting system to carry out uterine rings from patient cells. Souza et al. [43] obtained uterine rings of human myometrial cells. The cells were magnetized with biocompatible gold nanoparticles, iron oxide, and Poly-l-Lysine, which do not alter the behavior of cells. After a magnetization procedure, re-suspended cells were collocated under 384-well plates on the magnets to form tight ring structures per well. This fast patterning was used to study contractility of different inhibitors simultaneously with interesting results. Their multiple test in vitro showed differences in contractility response even when all the cells were from women. This fact demonstrates the differences between biological samples and the importance of personalized medicine in the near future as well as rapid patterning techniques.

**Figure 4:**
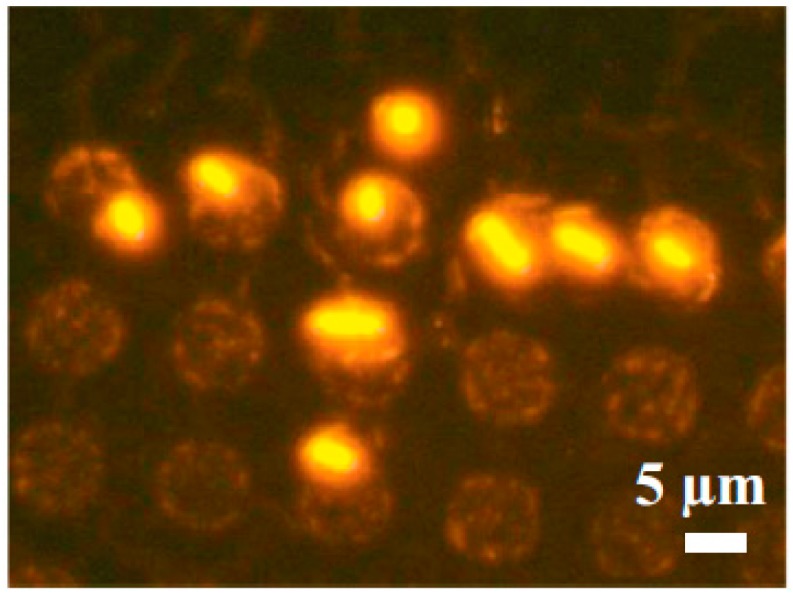
*E. coli* stained with ethidium bromide to observe individual cell-trapping on a micro-magnet array. Reproduced with permission from [44].

The necessity of single-cell studies of cell membrane functionality, the interaction with new drugs, the detection and sorting among other biological applications are now boosting single-cell arrays with magnetic approaches. Magnetic arrays are suitable for bacteria patterning despite the fact that bacteria cells are smaller than mammalian cells. Pivetal et al. [44] fabricated, by using reversed magnetization with thermomagnetic patterning, a patterned array of 7.5 × 7.5 mm^2^ micromagnets. Bacteria were labelled magnetically by immunomagnetic in situ hybridization to increase specificity and guarantee bacteria fixation (Figure 4). The above paper reported that both labelling techniques and fixed bacteria conserved their membranes thus being suitable for further studies.

In this context, magnetic patterning is specific for mammalian and bacteria cells. However, the technique requires cell labelling with biocompatible magnetic particles.

### 2.2. Chemical Patterning for Cells Assembly

#### Surface Chemistry Methodologies

Cells also have the ability to sense the environment around them, especially the surface where they are adhered. It is thus possible to take advantage of this property to pattern adhesive and anti-adhesive molecules and therefore order the cells on a surface. Proteins from the extra cellular matrix, like fibronectin, laminin or collagen, are preferred to glass or Poly(l-lysine)-graft-poly(ethylene glycol) (PLL-g-PEG) by the cells. This makes it possible to control the localization of the cells. Moreover, patterning special forms of adhesive molecules has been performed. Disc, crossbow, H, Y, L and many more were created and are commercially available for fundamental research in cancer, cell adhesion, architecture or mechanotransduction [45,46,47,48]. 

This technique has been used by Théry et al. [47], to better understand the role of the adhesive microenvironment and of the cell internal organization on the polarity. They demonstrated that the microtubule distribution, the position of the nucleus, centrosome and Golgi apparatus, depend on the shape of the printed ECM proteins. Thanks to this approach they established a link between the extracellular adhesion, the organelles organization, and the cell polarity. In the same study, the concept of the average cell was proposed. Fluorescence coming from different dyes is collected and several cells immobilized on the same sort of pattern. The pattern has the advantage of imposing a shape to the cells. Thus, combining several fluorescence images of several cells is possible. This was strictly impossible with cells freely sticking on a surface, because, each cell would take a different shape. There is a clear statistical interest to pattern and create arrays of cells having the same shape.

**Figure 5:**
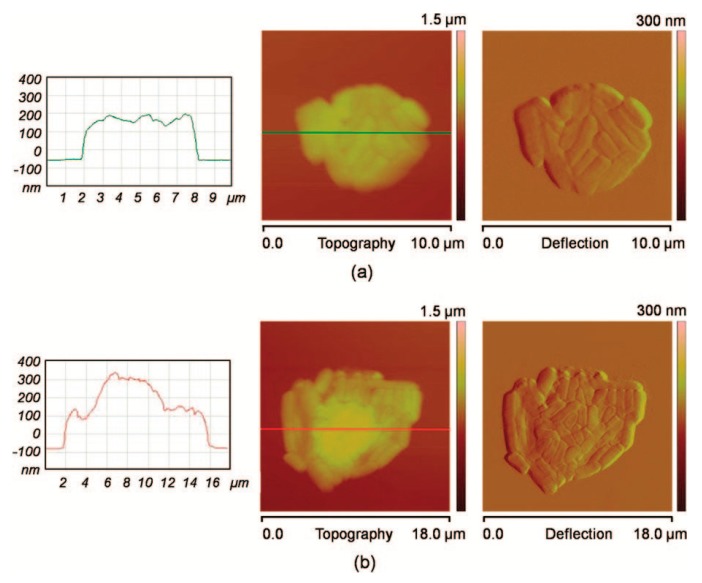
*E. coli* bacteria onto SiO_X_ patterns, observed by atomic force microscope (AFM) in contact mode: meniscus dragging speeds of (**a**) 1 µm/s and (**b**) 0.5 µm/s. Reproduced with permission from [54].

In microbiology, matrix proteins have not been used to immobilize bacteria or yeast. On the contrary some work relies on electrostatic interactions between a positively charged surface and negatively charged microbes. Polyethylenimine (PEI), Poly-l-Lysine (PLL) or 3-aminopropyltriethoxysilane (APTES) were used to immobilize microbes [49,50,51,52,53]. Only recently have researchers been interested in patterning positive charges to create bacteria arrays. In 2008, Ressier et al. [54] used Atomic Force Microscope (AFM) oxidation lithography to create patterns of SiO_X_ on a hydrophobic Self Assembled Mono-lay (SAM) of octadecyltrimethoxysilane (OTMS). The convective/capillary technique to direct the assembly of *E. coli* cells on the SiOx pattern was used (Figure 5). A more uniform pattern was achieved.

**Figure 6:**
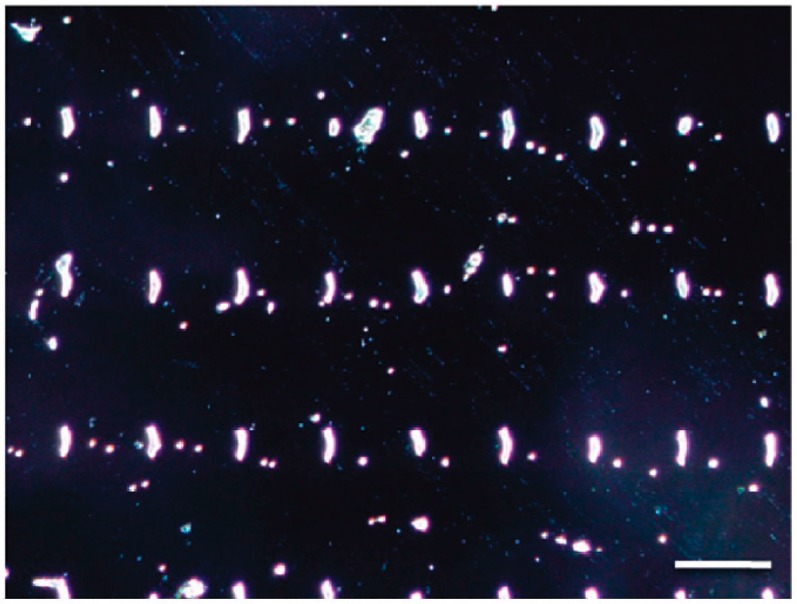
Patterned bacteria onto functionalized surface with 3-aminopropyltriethoxysilane (APTES) (1100 × 1000 μm^2^ dark field image with a scale bar that measures 30 μm). Reproduced with permission from [55].

In another study, Cerf et al. [55], used micro contact printing to create an anti-adhesive sea made of octadecyltrichlorosilane (OTS) and organized positively charged islands made of APTES. Thanks to this development they were able to assemble arrays of *E. coli* cells that were analyzed by AFM nanomechanical experiments. Cells killed by heating, were found to be stiffer than normal cells deposited on the pattern. Figure 6 shows the array of bacteria prepared on the bifunctional (adhesive: APTES positive charges and anti-adhesive: OTS) surface.

**Figure 7:**
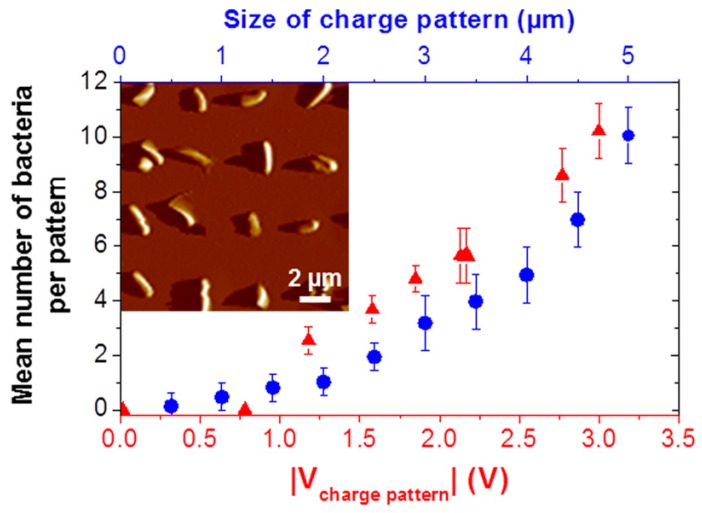
AFM deflection image, in a buffer medium, of an arrays of single *Pseudomonas aeruginosa* bacteria immobilized on polyethylenimine (PEI) patterns, with the lateral size (blue disk symbols) and charge patterns (red triangle symbols). Reproduced with permission from [56].

More recently Jauvert et al. [56], used high molecular weight PEI molecules to create a pattern of positive charges. In this work, negative patterns are created on a PMMA thin film, by nanoxerography or electrical micro contact printing. The surfaces were then immersed in PEI solution, and finally dried after a final immersion in ethanol. The PEI thickness is controlled by the amount of charge injected during the nanoxerography process, resulting in a control of the positive charge on the pattern and finally on the number of bacteria immobilized on each pattern. Figure 7 shows this dependency. 

### 2.3. Physical and Chemical Patterning

#### 2.3.1. Microcontact Printing Overview

Microcontact printing (µCP) is an accessible lithography technique first introduced by the Whiteside group [57]. It relies on a stamp made of an elastomeric material usually polydimethylsiloxane (PDMS) cast on a master mold (usually silicon). The unmold stamp is inked and let to dry. Finally, the ink is transferred onto a surface by contact. The contact is said to be conformational as the stamp elastic properties allow it to make conformal contact even on rough surfaces resulting in a high-quality transfer of the ink onto the surface. Although microcontact printing can be used to produce nanoscale patterns down to 40 nm line grating [58] and even 2 nm using nanotubes to mold the stamp [59], nano-lithographic facilities are required and are quite expensive and not necessary for cell adhesion. Microcontact printing is often presented as an accessible technology necessitating only a simple laser printer, spin coating and UV lamp to perform rapid prototyping of master molds [60] with features larger than 20 µm largely sufficient for cell patterning. Features larger than 2 µm have since become easily affordable as master molds can be ordered from specialized companies for a few hundred dollars and can then be used to produce an unlimited number of PDMS stamps. Since the first publication revealing cells attached to surface patterns using µCP [61], several reviews on microcontact printing have addressed in part the use of microcontact printing to attach cells onto surfaces [62,63,64]. Here we highlight some of the recent advances that have been made in the different steps involved in the µCP process: mold, inking process, stamping process or stamped surface/material. 

Fabricating a master mold is both expensive and time consuming. One way to avoid breaking the original silicon master mold is to make replicas in epoxy or polyurethane using PDMS stamps made from the original mold [65]. Fabricating a mold is often time consuming as it requires time to design it, instead why not use natural materials to produce patterns? Wong et al. [66] have used the vascular system of a leaf to produce a bioinspired PDMS mold. This mold was successfully used to grow endothelial cells into vascular channels. 

Often when considering technical options offered by µCP, the choice of ink seems the most viable. Which molecule will attach best to the cells used? What influence will the ink molecules have on the attached cells? Which molecule will prevent attachment outside the defined patterns? (These questions have mostly been answered in previously cited reviews.). Yet, cells are seldom considered as the actual ink. Malaquin et al. [67] used an inking technique derived from capillary assembly where a meniscus displacement was used to push and capture particles into grooves at the surface of a PDMS stamp. Capillary assembly has also been used to improve inking of molecules onto PDMS stamps resulting in much improved prints [68,69], using specific antibody coated at the surface of the particles and targeting cell membrane proteins. Delapierre et al. were able to capture and place specific types of cells onto the stamp [70]. Alternate grooves at 90° angle to each others allowed the alternate capture of particles coated with different antibodies capable of attaching two different cell types on the stamp. 

Automation [71] and robotics [72,73] have improved the robustness of the printing process. These commercial systems can align prints with sub-10 µm precision in a repetitive fashion and several different molecules can be printed. This opens the path to more complex devices with different specific cells at specific positions. However, the robustness of the printing process is dominated by the interaction between the ink and the surface. Humidified Microcontact printing is a new process for printing biomolecules susceptible to attach cells onto low energy surfaces such as plastic Petri-dishes. In this respect Ricoult et al. [74] showed that flowing water in channels next to proteins at the contact area between the stamp and the surface, improves the quality of prints on both low and high energy surfaces while increasing the distance from the water channel decreases print quality. Relative humidity at 88% in the stamp was found to be the threshold to increase the transfer of proteins and the overall robustness of the printing process. 

If glass and plastic Petri-dish surfaces are more commonly used for µCP, improvements in µCP can come from the surface on which molecules or cells are immobilized. Polio and Smith [75] developed a methodology to perform µCP on poly-acrylamide hydrogels to study 2D cellular traction forces. They advantageously used a microcontact printed coverslip to transfer alternate patterns of gelatin and fibronectin onto the surface of the hydrogel. Gels and scaffolds are increasingly being used for tissue engineering and to understand cells and tissue mechanobiology. An example of such an experiment is illustrated in the study by Vedula et al. [76] which shows that epithelial bridges between cells separated by tracks maintain tissue integrity during cell migration. In this case microcontact printing was performed to print fibronectin on top of non-adherent polymer tracks. A suspended membrane formed between the tracks as collective cellular migration took place. This last example illustrates how microcontact printing has become a versatile accessible technique for biology.

The use of PDMS based stamps in biology is not limited to microcontact printing and has also led to a new form of cell patterning using physical capture through microwells and using microfluidic devices that are shown in the following sections.

#### 2.3.2. Microwells and Filtration

**Figure 8:**
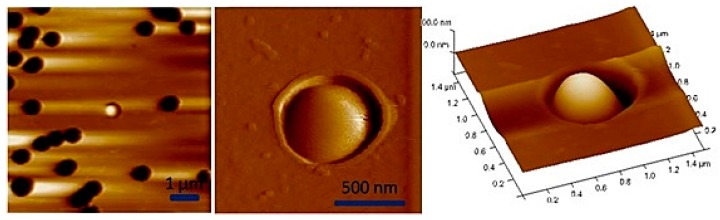
AFM images of the immobilization of *Lactococcus lactis* cell in pores of polycarbonate membranes (provided by Etienne Dague).

In an attempt to minimize the surface chemistry, microstructured surfaces or used porous membranes to immobilize round shaped cells have been developed. Figure 8 presents a single *Lactococcus lactis* cell trapped in a pore of a polycarbonate membrane. This technique has been extensively used to trap bacteria and yeast for AFM experiments [77].

**Figure 9:**
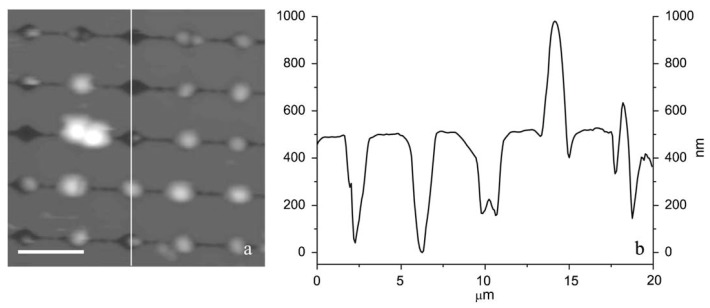
AFM image of *S. aureus* cells trapped in holes elaborated by contact mask photolithography and their variation in height measured by AFM. Reproduced with permission from [78].

Unfortunately, the filling rate of the pores is often very low, and it is time consuming to localize a cell and perform statistically relevant experiments. In order to overcome this problem Kailas et al. [78] developed a lithographically patterned substrate to immobilize *Staphylococcus aureus* cells (Figure 9). In this method no chemicals are used to immobilize the cell and the confinement is minimized as compared to the filter solution because no sucking step is performed. However, an evaporation step of 15 to 20 min is required to allow the cells to settle into the patterns. Thanks to this device, it was possible to follow the cell division process under AFM.

**Figure 10:**
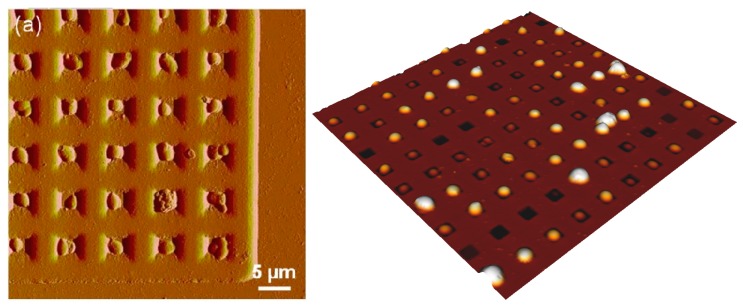
AFM images of *S. cerevisiae* yeast trapped in polydimethylsiloxane (PDMS) patterns functionalized by Concanavalin A (on the left) reproduced with permission from [79] and AFM 3D height image of *C. albicans* cell array trapped in microwells made of PDMS stamps (on the right). Reproduced with permission from [15].

Furthermore, Dague et al. [15,79] developed a microstructured PDMS stamp presenting various holes size, ranging from 1.5 × 1.5 µm^2^ to 6 × 6 µm^2^. The stamp is prepared by molding PDMS on a silicon master with negative patterns (Figure 10). The silicon master is elaborated by performing conventional photolithography and reactive ion etching. The authors demonstrated that the PDMS stamp is suitable for immobilizing not only bacteria and yeasts, but also algae and eukaryotic cell nuclei [15]. To achieve a higher filling rate of the hole, the authors took advantage of convective/capillary deposition and achieved a filling rate of up to 85%. Such a development is a step toward the fabrication of reproducible microbial cell arrays where each cell can be probed individually. Thanks to such developments, it will be possible in the near future to access the population heterogeneity, which is known to be a key factor in bacterial resistance acquisition, for instance.

#### 2.3.3. Cell Patterning in Microfluidic Devices Combined with Microcontact Printing

**Figure 11:**
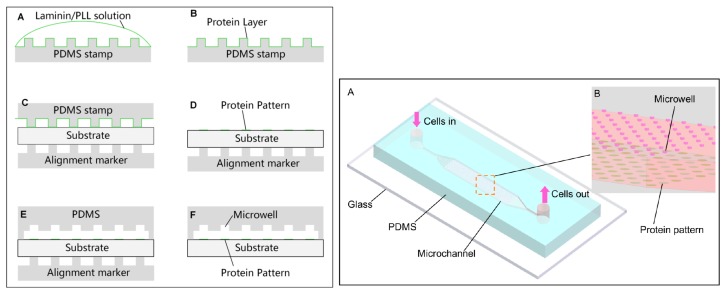
On the left, a schema of a PDMS stamp with microwells to fabricate the microfluidic device, showing protein incubation (**A**,**B**), patterned proteins on the substrate (**C**,**D**) and microwell paring with a PDMS alignment marker. On the right, a schema of the final microfluidic chip showing triangular microwells where cells are captured. Reproduced with permission from [82].

Micropatterning cells inside microfluidic devices has enormous research application; to implement 3D culture of a specific cell line for instance and then to study cell signaling, proliferation or cell migration. In this context, a method to pattern cell culture inside a microfluidic device was reported [80] in which success was achieved in implementing the binding and sterilization, in one step, of human umbilical vein endothelial cells (HUVEC), MDA-MB-231 breast cancer cells, and NIH 3T3 mouse fibroblasts. As it is a physicochemical patterning methodology, a substrate with PLL, collagen, and other extracellular matrix (ECM) proteins (cell-adhesive) was functionalized, by using microcontact printing (µCP) and the plasma-based dry etching process to bond and etch away some parts that were not in contact with the PDMS, to finally integrate a PDMS-based microchannel piece and to complete the microfluidic device. In a more recent article [81], a microfluidic cell patterning method was developed to form patterned 3D multicellular aggregates (spheroids) of multiple cell types. This device was composed of one top PDMS channel, sandwiching a semi-porous polycarbonate membrane and a bottom PDMS channel, so that the flow and cells pass through them. Finally, the group of Xuesong Ye et al. [82], in a very recent experiment, developed a microfluidic chip to pattern two cancer cell lines; HeLa and human gallbladder carcinoma cells (SGC-996) and were able to observe phenomena such as colony formation, cell migration, and cell proliferation. Firstly, PLL and Laminin proteins were printed with µCP and then a PDMS stamp, carrying paired microwells, was incubated on the substrate of the microfluidic chip (Figure 11). They employed a SU-8 photolithographical process to elaborate the different utilized pieces to finally implement cell patterning in 5 min, the loading of cells was performed by a syringe pump.

#### 2.3.4. Deep UV Micropatterning

This methodology applies wavelengths of below 280 nm in the region of deep UV (DUV) to obtain micropatterns, it requires a predesigned photomask sensitive to those wavelengths. The material used in a photomask especially for deep UV is normally natural quartz, synthetic quartz or fused silica [83]. However, this technology has also been used for glass and PDMS combined with a coating of PLL and PLL-g-PEG to facilitate cell adhesion [84,85]. Alvéole PRIMO^®^ technology [86], based on light induced molecular adsorption of proteins (LIMAP), enables protein micropatterning to adhere specific cells. The photoactivable reagent is exposed to UV light (PRIMO module) to obtain patterns of up to 1.2 µm resolution. This approach facilitates the manipulation and elaboration of cell arrays for measuring and it is possible to combine this technique with conventional processes.

Hulshof et al. [87] used deep UV lithography in combination with conventional lithography to fabricate more than 1200 different nanotopographies for cell cultivation. U2OS osteosarcoma cells were cultured in their chip to measure cell spreading, orientation, and actin morphology in their topography designs which include lines, circles, and triangles in different arrays of 200 nm to 700 nm. They observed relevant changes in cell behavior related to their topographies.

This technology is mainly used by biologists because it does not require expensive facilities to perform the process and it is a better method for cell manipulation. 

## 3. Perspective: Automatic Biophysical Measurements on Patterned Cells

Based on the variety of techniques to pattern different kinds of cells, it seems that the next step is to perform biophysical measurements on them automatically (e.g., mechanical, force distribution). At present only a few researchers are taking advantage of cell arrays to develop automated systems. For instance Li et al. [88] designed a nanomanipulator to integrate it in an Atomic Force Microscope (AFM). They tested this system analyzing lymphoma Raji cells. Pillars (5 µm in height and 10 µm in diameter) were elaborated and then coated with Poly-l-Lysine (PLL) so the lymphoma cells were vertically trapped. Another example was published by Fortier et al. [89] where microwells were elaborated onto a glass coverslip coated with SU-8 film using a soft lithography technique to obtain an array of circular wells (20 µm in diameter, 7 µm in height). Then, the mechanical properties of fixed leukemia cells (NB4) were measured, implementing an automated system for the data analysis; their software processed 147 force curves taken at different applied forces with the objective of determining which geometry tip (spherical or conical) was more convenient for NB4 cells. Eleonora Minelli et al. [90], recently reported a fully automated neural network based algorithm to analyze 200 approach/retract force-distance (FD) curves, taken by AFM, applied to brain cancer tissues. In this respect, we are developing a methodology which permits us to obtain and analyze automatically thousands of biophysical measurements of both mammalian and bacterial cells, in a few hours.

## 4. Conclusions and Perspective

Physical and chemical micropatterning techniques have improved rapidly and several methodologies are emerging. The selection of the best technique will depend primarily on the purpose as well as the biomaterials involved, the experimental design, and the micro-nanofabrication techniques. On the one hand, physical methods for cell trapping such as inkjet printing, optoelectronic, acoustic, dielectrophoretic, laser-based, and magnetic techniques provide high specificity to sort and collocate cells in predesigned patterns. This may simplify further tests and considerably reduce costs, the amount of material used and biological samples for high-throughput analysis. However, with these methods collateral effects on cells such as opticution, poration, or cell damage can appear because of the thermal effects caused by external energy sources. These physical-active techniques can be efficient, highly specific and reproducible, but it is necessary to identify the critical factors for each technique (Table 1), to conserve viability and cell functionality.

On the other hand, the use of surface chemistry based methodologies provide an efficient way to fix cells on surfaces taking advantage of biomolecule specific recognition by cell receptors and chemical bonding between different functional groups which allow high adhesion, specificity or the opposite effect such as repelling adhesion. Micropatterning techniques such as microcontact printing have extended their applications even in the microfluidic area and novel in vitro models with patterned cells are increasing and impacting on future studies related to intracellular sensing; 3D portable in vitro models for diagnosis and therapy, used in point-of-care (POC) biomedical devices. All these technological advances have greatly expanded the development of biomedical microdevices and high-performance platforms to automatically analyze cells as medical applications are emerging, with great academic and industrial impact.

## Figures and Tables

**Table 1:** Advantages and disadvantages of cell patterning and manipulation techniques.

Technique	Advantage	Disadvantage
Inkjet printing (Physical)	Moderate cost Good controllability	Droplet formation Requires an external power source
Optical and optoelectronic cell trapping (Physical)	Remote and large-scale manipulation Highly specific due to the intrinsic charge and dielectric properties of cells	Thermal effects and photodamage in cells Requires an external power source
Laser-based cell patterning (Physical)	Cells and any particles can be manipulated	Large instrumentation Complex set-up
Acoustic patterning (Physical)	No significant heat generation and no effects on cell viability	Requires an external power source, piezoelectric surface, and electrode fabrication.
Dielectrophoresis (Physical)	Combine electrokinetic forces with hydrodynamic effects High-resolution patterning Large-scale parallel manipulation	Requires an external power source Dielectric force decreases due to the separation distance of electrodes
Magnetic techniques (Physical)	Remote manipulation High-resolution patterning, No stress behavior on cells	Magnets and labelling cells with magnetic particles are required
Surface chemistry methodologies (Chemical)	High precision and recognition by receptor or specific functional groups between the surface and cells	Pretreated surface is required The surface chemistry could modify the functionality of cells
Microcontact printing (Physicochemical)	Low cost, rapid prototyping	Difficulty in controlling the ink and the surface robustness
Microwells and filtration (Physicochemical)	Minimize the surface chemistry and conservation of cell functionality	Time consuming placing numerous cells inside microwells
DUV patterning (Physicochemical)	It does not require expensive facilities	The resolution depends on the photomask design and patterning substrate.
Cell patterning in microfluidic devices combined with microcontact printing (Physicochemical)	Study 3D culture cells and specialized biomedical microdevices	Requires specialized facilities, integration of techniques
